# Isolation and Identification of Major Pathogenic Bacteria from Clinical Mastitic Cows in Asella Town, Ethiopia

**DOI:** 10.1155/2020/6656755

**Published:** 2020-11-13

**Authors:** Gezehagn Kasa, Betelihem Tegegne, Belege Tadesse

**Affiliations:** Wollo University School of Veterinary Medicine, P.O. Box 1145, Dessie, Ethiopia

## Abstract

Mastitis is a multietiological and complex disease causing inflammation of the parenchyma of mammary glands and is a problem in many dairy cows. The objective of this study was to isolate and identify the pathogenic bacteria that cause bovine clinical mastitis. A cross-sectional study was undertaken between November 2018 to April 2019 on a small scale and government dairy farms in Asella town. Cow's udder and teats were physically examined to detect clinical mastitis. A total of 83 milk samples were collected from 46 cows that show clinical sign of mastitis from a total of 12 farms. Isolation and identification of major bacterial species were carried out by culturing different media and using primary and secondary biochemical tests. Out of the 83 samples collected and examined, all (100%) were positive for the cultural isolation of bacterial species. The bacteria were identified to genus and species level. Among the 83 isolates, 32 (38.6%), 24 (28.9%), and 6 (7.2%) were *Staphylococcus aureus*, *Staphylococcus intermedius,* and *Staphylococcus hyicus,* respectively. Other bacteria like *Escherichia coli* 12 (14.5%) and *Streptococcus* species 2 (2.4%) were also isolated. *Bacillus* species 2 (2.4%), *Proteus* species 2 (2.4%), and 3 (3.6%) of them were mixed bacterial infections. The present study revealed that both contagious and environmental bacterial pathogens were responsible for the occurrence of clinical mastitis. Proper milking practices and farm husbandry practices and future detailed studies up to the species level and on antibiotic profiles of the pathogens are needed.

## 1. Introduction

Ethiopia is believed to have the largest livestock population in Africa [1]. The total cattle population in the country is estimated to be about 56.71 million. Out of this, the female cattle constitute about 55.45%, and the remaining 44.55% are male cattle. From the total cattle 98.66% in the country are local breeds and the remaining are hybrid and pure exotic breeds that accounted for about 1.19 and 0.14%, respectively [1]. The livestock sector has been contributing a considerable portion to the economy of the country and still promising to rally round the economic development of the country. However, milk production does not satisfy the country's requirements due to a multitude of factors [2]. Mastitis is among the various factors contributing to reduced milk production. Bovine mastitis is the second most frequent disease next to reproductive disorders and one of the major causes of economy failure in Ethiopia. It affects both the quantity and quality of milk [3].

Mastitis is a multietiological and complex disease, which is defined as inflammation of the parenchyma of mammary glands [4]. The disease mainly resulted from injurious agents including pathogenic microorganisms, trauma, and chemical irritants. Even if it occurs due to the injury of any type, the udder disease of major concern is that associated with microbial infection. Among various infectious agents, bacterial pathogens have been known to be widely distributed in the environment of dairy cows, constituting a threat to the mammary gland [5]. Over 130 different microorganisms have been isolated from mastitis positive cow milk samples, of which almost all are bacteria. The most common pathogens comprise contagious bacteria, mainly *Staphylococcus aureus* and *Streptococcus agalactia* and environmental bacteria mainly coliforms and some species of streptococci that are commonly present in the environment [5, 6].

Mastitis can be manifested by a wide range of clinical and subclinical conditions. Clinical mastitis is characterized by sudden onset, alterations of milk composition and appearance, decreased milk production, and the presence of the cardinal signs of inflammation in infected mammary quarters. It is readily superficial and visually detected. It occurs when the inflammatory response is strong enough to cause visible changes in the milk (clots and flakes), the udder (swelling), or the cow (off feed or fever). Even if there is a great loss related to both conditions, clinical mastitis continues to be a problem in many dairy herds [7, 8].

Mastitis is a global problem as it adversely affects animal health, quality of milk, and the economies of a country by causing huge financial losses [9]. There is agreement among authors that mastitis is the most widespread infectious disease in dairy cattle and, from an economic aspect, the most damaging [10]. This disease has also been known to cause a great deal of loss or reduction of productivity, to influence the quality and quantity of milk yield and to cause culling of animals at an unacceptable age. Most estimates have shown a 30% reduction in productivity per affected quarter and a 15% reduction in production per cow/lactation, making the disease one of the costliest and serious problems affecting the dairy industry worldwide [8]. Clinical mastitis in a dairy herd is threatening to a farmer, but treatment can be given immediately to control it [7]. Mastitis is worth studying as it incurs financial losses attributed to reduced milk yield, discarded milk following antibiotic therapy, early culling of cows, veterinary costs, drug costs, increased labor, death in peracute septicemia, and replacement cost [11].

In Ethiopia, different studies show the prevalence of the cause of clinical mastitis in different parts of the country. For example, in Harrarghe Zone, the predominant isolated bacteria were coagulase negative *Staphylococcus* species (CNS) (34.2%) followed by *Staphylococcus aureus* (24.2%) [12]. In other studies, the predominance of *coagulase negative Staphylococcus* (43.47%) and *S. aureus* (36.95%) was also reported in a study conducted in other parts of the country [13–16]. In a study conducted in Bishoftu town, the major pathogens isolated were *Staphylococcus aureus* (44.95%), *S. intermedius* (22%), *S. haicus* (9.2%), other *Staphylococcus* spp. (23.9%), *Streptococcus* spp. (28.1%), and *E. coli* 9.8% [17]. Moreover clinical mastitis is a frequently occurring and economically important disease for the dairy industry in our country, Ethiopia. For the control and prevention of the disease, proper isolation and identification of the responsible bacterial agents are necessary regarding which little studies are still done in the current study site. Therefore, this study was done to isolate and identify pathogenic bacteria from cows that have clinical mastitis.

## 2. Materials and Methods

### 2.1. Study Area

The study was conducted between November 2018 and April 2019 in Asella town. The town is located in Arsi Zone of Oromia region about 175 km from Addis Ababa. The town has a latitude and longitude of 7°57′N and 39°7′E, with an elevation of 2,430 meters above sea level. Topographically Asella is a highland area with an annual rainfall of 2300 to 2400 mm [18].

### 2.2. Study Animals

Lactating dairy cows found in privately owned small holder dairy farms and government dairy farms in Asella town were involved in the study population. The study was conducted on purposely selected lactating dairy cows with clinical signs of illness regardless of the age, breed, pregnancy, husbandry system, hygienic condition, milking practice, parity, and stage of lactation.

### 2.3. Study Design and Sampling

A cross-sectional study that involved laboratory isolation and identification of bacteria was undertaken from November 2018 to April 2019 on a small scale and government owned dairy farm in Asella town. Cows that showed signs of clinical mastitis were selected and sampled purposively from the farms that are found in the town (all dairy farms found in the town were included in the study). A total of 83 milk samples from 44 dairy cows that have shown clinical signs of mastitis during the study period from 12 dairy herds were sampled.

### 2.4. Data Collection

#### 2.4.1. Questionnaire Survey

The data about the factors like age, breed, pregnancy, parity number and lactation stages of the cows and milking practice, husbandry system, and hygienic condition of the farms were collected from cattle owners and farm managers through a face to face questionnaire survey.

#### 2.4.2. Physical Examination of Udder and Milk

Cow's udder and teat were examined for the signs of clinical mastitis. The udders of the study cows were examined visually and by palpation for the presence of clinical mastitis. During examination, attention was given to cardinal signs of inflammation (i.e., redness, swelling, pain, hotness, and loss of function), size and consistency of udder quarters. Inspection of milk for discoloration, consistency, and presence of clots, which are characteristics of clinical mastitis, was performed.

#### 2.4.3. Milk Sample Collection

A total of 83 milk samples were collected from 46 cows which show clinical signs of mastitis in Asella town from a total of 12 small scales and government owned dairy farms. The milk samples were collected from the teats of clinically infected quarter's from cows that are not treated early with either intramammary or systematic antimicrobial agents. Milk sampling was carried out following aseptic procedures as described by the National Mastitis Council [19].

#### 2.4.4. Collection, Transportation, and Storage of Milk Samples

The udder was first washed with water and then the teats and teat orifices were disinfected with pieces of cotton wool soaked in 70% ethyl alcohol and then dried with fresh pieces of cotton wool. Approximately 5-6 ml of milk from the infected quarter were taken (after discarding the foremilk) aseptically in sterile bottles for bacteriological investigation and labeled accordingly. Samples were placed in ice box containing ice packs and transported immediately to the microbiology laboratory in the Asella Regional Veterinary laboratory. Samples that are not processed immediately were preserved in a refrigerator at 4°C until processing (until 48 hours).

### 2.5. Laboratory Analyses

#### 2.5.1. Bacterial Isolation

For the isolation of bacteria, different types of media were used (solid and liquid media). The common media used during the study were blood agar, nutrient agar, MacConkey agar, Mannitol salt agar, Eosin methylene blue medium, nutrient broth, Triple sugar iron agar, Simon citrate agar, Tryptophan broth, and MR-VP broth biochemical media were used. The media for the laboratory analysis were prepared according to standard procedures recommended by Quinn et al. [6].

#### 2.5.2. Cultural Methods

The samples collected from cows were cultured on general purpose media such as blood agar and nutrient agar using a sterile loop inside the biosafety cabinet and around the Bunsen burner. Other selective and differential media such as Mannitol salt agar, MacConkey agar, Eosin methylene blue agar were also used for cultural purposes. Inoculated plates were incubated aerobically at 37°C. After 24 hours of incubation, the plates were removed from the incubation and examined visually. Any growth, pigmentation, hemolysis, and colonial morphology were noted accordingly.

#### 2.5.3. Identification of Isolates Using Gram Stain Reaction and Biochemical Tests

Colonies representative of each type of bacterium were stained by Gram's method and then examined microscopically for Gram staining reaction (positive staining purple or negative staining pink), size (small, medium, or large), and shape (rods, cocci, or coccobacilli). Further characterization of the isolates was done using conventional biochemical tests (catalase, oxidase, indole production, methyl red test, Voges–Proskauer test, citrate utilization, triple sugar iron, and coagulase tests) following Markey et al. (2013).

### 2.6. Data Management and Analysis

The data including the quarter affected, parity number, husbandry system, hygienic condition, lactation stage, and milking practice were recorded depending on clinical inspection; pathogenic bacteria isolated and identified were entered into Microsoft Excel computer program 2007. STATA version 14 was used to summarize the data, and descriptive statistics like percentages were used to express the result.

## 3. Results

### 3.1. Prevalence of Bacteria Isolated from Mastitic Milk

In the current study, all (100%) the 83 milk samples collected from clinically mastitic cow were positive for the cultural isolation of bacterial species. The bacteria were also identified to genus and species level. Most isolates were *Staphylococcus* species 62 (74.7%), in which all of them were coagulase positive. Among the total of 83 isolates, 32 (38.6%) were *Staphylococcus aureus*, 24 (28.9%) were *Staphylococcus intermedius,* and 6 (7.2%) were *Staphylococcus hyicus*. Other bacteria like *E. coli* 12 (14.5%) and *Streptococcus* species 2 (2.4%) were also isolated (Table 1).

#### 3.1.1. Proportion of Clinical Mastitis at Teat Level

There was no significant difference between quarters in the occurrence of pathogenic microorganisms (*p* > 0.05). However, the highest proportion of microorganisms has been isolated from left back teat, 25 (30.1%). From all teats except for the left front, the highest proportion has been recorded in *Staphylococcus aureus.* Mixed infection was found on the left front and right back quarter (Table 2).

### 3.2. Description of Study Farms and Animals

The study was performed in 12 farms and 10 (83.3%) farms were managed intensively and the other 2 (16.7%) were semi-intensive farms. From the total number of farms in which the study was conducted, 6 (50%) of the farms were managed in a poor hygienic condition. From the farms included in the study, 11 (91.2%) farms kept only cross breed cows, whereas only one farm kept local breed cows (Table 3). The profiles of cows included in the study were shown in Table 4.

## 4. Discussion

A total of 83 milk samples were collected and processed from clinically infected cows from small scale holder and government dairy cows in Asella town. The result of the current study showed that *Staphylococcus* species, *Streptococcus* species, *Escherichia coli*, *Bacillus* species, and *Proteus* species were isolated which has also been reported in another study [20].

Isolation and identification of pathogenic bacteria such as 38.6% *S. aureus*, 28.9% *S. intermedius*, show the high contributions of microbial agents as a cause of mastitis in the area. From coagulase positive *Staphylococcus* species, the predominant pathogen isolated in the current study was *S. aureus* (38.6%). This finding is in agreement with 39.1% reported by Bedada and Hiko [21] whereas higher than the report of Mulugeta and Wassie [22], who reported an isolation rate of 30.0%. The reason for higher isolation rates of *S. aureus* is its wide ecological distribution inside the mammary gland and skin. In areas where hand milking and improper use of drug is practiced to treat mastitis case, its dominance has been suggested and might be due to the fact that they are easily transmitted during milking via the milker's hands as they are contagious pathogens [23].

Laboratory results from the current study indicated that the prevalence of *Staphylococcus intermedius* was 28.9%, which are the second predominant isolated bacteria next to *Staphylococcus aureus*. The result reported from the current study was lower than the (38.4%) reported by Argaw and Tolosa [24] but much higher than reports of Birhanu et al. [25], who reported 7.14% in the same study site. The variability in the prevalence of isolated bacteria between reports could be attributed to differences in the management of the farms, milking practices, and hygienic condition of the farms [26].

The 14.5% isolation rate of *E. coli* found in this study was comparable with the findings of Demme and Abegaz [20], who reported 18.6% at Addis Ababa, while it was found to be lower than the 40.7% reported by Iqbal et al. [27] and much higher than the report of Birhanu et al. [25], who reported 5.71% in different parts of Ethiopia. The prevalence of environmental *E. coli* may be associated with poor farm cleanliness and poor slope of stable areas. Feces which are common sources of *E. coli* can contaminate the premises directly or indirectly through bedding, calving stalls, udder wash water, and milker's hands [5].

The 2.4% proportion of *Streptococcus* species found in this study was much lower than the findings of Demme and Abegaz, [20] who reported 16.7% *Streptococcus* species. The variability in the prevalence of isolated *Streptococcus* species between reports could be because of some contagious *Streptococcus* species survives poorly outside the udder, and established infections are eliminated by frequent use of penicillin and other antibiotics and because of difference in the milking practice between the different farms in the studies [5].

The results from the current study indicated that the proportions of *Bacillus* species were low (2.4%) which was similar to the findings of Bedada and Hiko [21] who reported 3.4% proportion. *Bacillus* species are only occasionally mastitis causing pathogens. The infection is associated with contamination of teat.

The 2.4% *Proteus* species isolated was almost similar to a 2.63% report of Hussein [28] in and around Addis Ababa and 2.2% report of [26]. The prevalence of *Proteus* species might be due to the residing of this agent in the cow's environment bedding, feed, and water. They spread due to poor environmental sanitation and milking practice.

The current study revealed that clinical mastitis has affected cows at different stages of lactation, early (41.3%), mid (32.6%), and late (26.1), which was comparable with the finding of Kerro and Tareke, [29] who reported a high prevalence rate of clinical mastitis of the cow in early lactation.

The occurrence of mastitis for cows that give birth for 3^rd^ and 4^th^ times was 26.09%, which was lower than the findings of Demme and Abegaz [20], who reported a prevalence of 71.5% during the 3^rd^ and 4^th^ parity. In this study, during midparity number, a high proportion was recorded. This could be associated with the possibility of exposure to the infectious agent with an increasing number of parities. This was in agreement with the findings of Biffa et al. [2] and Tesfaye [30]. Again, it also agreed with the report of Demme and Abegaz [20], who report high proportion during the medium of parity and when reaching 5^th^ parity number it reduces; this is due to the farm management system, culling of too old lactating cow and there is a small number of cows giving birth for fifth (5^th^) times and more.

## 5. Conclusion and Recommendations

The present study which was conducted on isolation and identification of major bacterial pathogens from clinically mastitic cows revealed that both contagious and environmental pathogens, such as *S. aureus*, *S. intermedius*, *S. hyicus*, *Streptococcus* species, *Bacillus* species, *Protes* species, and *E. coli* were isolated. From the isolated organisms, *S. aureus* (38.6%), *S. intermedius* (28.9%), and *E. coli* (14.5%) were the predominant organisms. This indicates that contagious mastitis is one of the major problems of dairy cows in milk production, followed by environmental mastitis. To reduce the problem of clinical mastitis, proper milking practices like milking of infected cows after milking of apparently healthy animals and regular cleaning of cow's udder should be practiced. Farm husbandry practices should be maintained to avoid contamination of cows' house and bedding to control and prevent environmental mastitis. There is a need for further detailed studies on different pathogenic microorganisms to the species level and antibiotic susceptibility pattern of those microorganisms that cause clinical mastitis.

## Figures and Tables

**Table 1:** Frequency and percentage of various bacterial species isolated from clinical mastitic samples (*n* = 83) from Assela, Ethiopia.

Bacterial species	Frequency	Percentage (%)
*Staphylococcus aureus*	32	38.6
*Staphylococcus intermedius*	24	28.9
*Staphylococcus hyicus*	6	7.2
*Escherichia coli*	12	14.5
*Bacillus* species	2	2.4
*Proteus* species	2	2.4
*Streptococcus* species	2	2.4
Mixed infection	3	3.6
Total	83	100

**Table 2:** Proportion/infection rate of clinical mastitis at the teat level.

Pathogen	Number of isolates (proportion)					
	Left front (LF)	Left back (LB)	Right front (RF)	Right back (RB)	Total	*p*-value
*S. aureus*	2 (6.2%)	11 (34.4%)	10 (31.2%)	9 (28.1%)	32 (100%)	0.078
*S. intermedius*	9 (37.5%)	5 (20.8%)	6 (25%)	4 (16.7%)	24 (100%)	
*S. hyicus*	3 (50%)	3 (50%)	0 (0%)	0 (0%)	6 (100%)	
*E. coli*	0 (0%)	6 (50%)	2 (16.7%)	4 (33.3)	12 (100%)	
*Bacillus* species	1 (50%)	0 (0%)	0 (0%)	1 (50%)	2 (100%)	
*Protes* species	0 (0%)	0 (0%)	1 (50%)	1 (50%)	2 (100%)	
*Streptococcus* species	1 (50%)	0 (0%)	1 (50%)	0 (0%)	2 (100%)	
Mixed infection	1 (33.3%)	0 (0%)	0 (0%)	2 (66.7)	3 (100%)	
Total	17 (20.5%)	25 (30.1%)	20 (24.1%)	21 (25.3%)	83 (100%)	

**Table 3:** Farm level description of factors.

Variable		Frequency	Percentage
Husbandry system	Intensive	10	83.3
	Semi-intensive	2	16.7

Hygienic condition	Poor	6	50
	Medium	4	33.3
	Good	2	16.7

Breed	Local (Borena)	1	8.3
	Cross	11	91.7

Total farms		12	100

**Table 4:** Profiles of cows included in the study.

Variable		Frequency	Proportion
Breed	Local (Borena)	2	4.3
	Cross	44	95.7

Age	Young	1	2.2
	Adult	39	84.8
	Old	6	13

Parity no	1	6	13.04
	2	9	19.57
	3	12	26.09
	4	12	26.09
	5	6	13.04
	6	1	2.18

Pregnancy	Pregnant	17	37
	Nonpregnant	29	63

Lactation stage	Early	19	41.3
	Mid	15	32.6
	Late	12	26.1

Total		46	100

## Data Availability

All the data analyzed during this study are included in this manuscript.
